# MtDNA-maintenance defects: syndromes and genes

**DOI:** 10.1007/s10545-017-0027-5

**Published:** 2017-03-21

**Authors:** Carlo Viscomi, Massimo Zeviani

**Affiliations:** MRC-Mitochondrial Biology Unit, MRC MBU, Wellcome Trust/MRC Building, Hills Road, Cambridge, CB2 0XY UK

## Abstract

A large group of mitochondrial disorders, ranging from early-onset pediatric encephalopathic syndromes to late-onset myopathy with chronic progressive external ophthalmoplegia (CPEOs), are inherited as Mendelian disorders characterized by disturbed mitochondrial DNA (mtDNA) maintenance. These errors of nuclear-mitochondrial intergenomic signaling may lead to mtDNA depletion, accumulation of mtDNA multiple deletions, or both, in critical tissues. The genes involved encode proteins belonging to at least three pathways: mtDNA replication and maintenance, nucleotide supply and balance, and mitochondrial dynamics and quality control. In most cases, allelic mutations in these genes may lead to profoundly different phenotypes associated with either mtDNA depletion or multiple deletions.

## Introduction

Mitochondria are essential organelles found in nearly all eukaryotic cells (Vafai and Mootha 2012), where they are mainly related to energy metabolism. The most prominent role for mitochondria is to generate ATP through the respiratory chain (RC) by means of oxidative phosphorylation (OXPHOS). However, mitochondria also take part in a vast array of important biochemical pathways including, among others, heat production, apoptosis, generation and detoxification of reactive oxygen species (ROS), intracellular Ca^2+^ regulation, steroid hormone and heme synthesis and lipid metabolism (Wallace 2005).

The RC is composed of four multiheteromeric complexes (CI-IV), which transfer the electrons extracted from nutrients by means of sequential redox reactions to molecular oxygen to form water. This process is coupled to the translocation of proton across the IM by three of the four canonical respiratory chain complexes (CI, CIII, and CIV), generating a proton gradient, which is then exploited by ATP synthase (or complex V, CV) to convert ADP into ATP.

Mitochondria are semi-autonomous organelles because they have their own DNA (mitochondrial DNA, mtDNA), which encodes for 13 essential subunits of CI, CIII, CIV, and CV (Schon et al 2012), whereas all the other components of the RC, and indeed all of the other ≈1500 polypeptides constituting the mitochondrial proteome, are encoded by the nuclear genome. Therefore, mitochondrial bioenergetics and related homeostatic and execution pathways are under the double genetic control of both nuclear and mtDNA. The genetic duality of mitochondrial metabolism has relevant consequences for human pathology, as mutations in either nuclear or mtDNA can lead to mitochondrial dysfunction and disease. These genetic features explain why mitochondrial disorders can be transmitted as dominant, recessive, X-linked or maternally-inherited traits (Zeviani and Di Donato 2004). Hundreds of pathogenic mtDNA mutations have been reported (MITO-MAP 2012). Whilst mtDNA in normal conditions is essentially uniform, a condition called homoplasmy, most of the pathogenic mutations of mtDNA co-exist with a variable percentage of wild-type mtDNA, in a condition termed heteroplasmy. Mutations of mtDNA or of OXPHOS-related nuclear genes can affect virtually every tissue in the body, and lead to different phenotypes depending on the organ involved, intrinsic severity of the mutation, targeted gene, and, in case of mtDNA mutations, heteroplasmy levels. Tissue and organ functions critically depend on adequate ATP production, especially when energy demand is high, like in neurons and muscle fibers (Koopman et al 2013). This explains why primary disorders of mitochondrial bioenergetics usually cause neurodegeneration and/or myopathy, often in combination, to determine encephalomyopathies affecting children or adults. However, many mitochondrial disorders also involve additional organs, e.g., causing heart impairment (for instance cardiac failure due to cardiomyopathy, or heart conduction defects), liver dysfunction, diabetes mellitus, or alterations in special senses or specific districts, e.g. sensory-neural deafness, ophthalmoparesis (due to weakness of the extrinsic eye muscles), ptosis (drooping eyelids), retinitis pigmentosa, and optic neuropathy causing blindness due to degeneration of the optic nerve. Mutations in mtDNA can affect specific proteins of the respiratory chain or the in situ, autochthonous synthesis of mitochondrial proteins as a whole (when mutations or deletions involve tRNA or rRNA genes) and can be in turn classified into large-scale rearrangements (i.e., partial deletions or duplications) and point mutations. Both groups have been associated with well-defined clinical syndromes. Whilst single large-scale rearrangements are usually sporadic, point mutations are tipically maternally inherited. Large-scale rearrangements include several genes and are invariably heteroplasmic. In contrast, point mutations may be heteroplasmic or homoplasmic, the latter being characterized by incomplete penetrance. (e.g., Leber’s hereditary optic neuropathy).

In addition to sporadic or maternally inherited disorders due to mutations of the mitochondrial genome, mitochondrial diseases can also be transmitted as Mendelian traits. Here, we shall focus on those Mendelian disorders that alter the stability and the integrity of mtDNA. In 1989, Zeviani and colleagues described an Italian family with adult-onset mitochondrial myopathy characterized by chronic progressive external ophthalmoplegia, CPEO, and inherited in an autosomal dominant fashion (Zeviani et al 1989). Maternal inheritance was excluded because the male patients also transmitted the disease to their offspring. Since then, many additional autosomal dominant CPEO families have been described. A second group of syndromes, characterized by infantile myopathy or hepatopathy, was then associated with depletion of mitochondrial DNA in affected tissues (Moraes et al 1991). Multiple deletions and depletion of mitochondrial DNA were also found in skeletal muscle in a complex, multisystem syndrome combining muscle, brain, and gastrointestinal symptoms (mitochondrial neurogastrointestinal encephalomyopathy or MNGIE) (Hirano et al 1998). Finally, mutations in the gene encoding the DNA polymerase gamma (*POLG*), the master enzyme of mitochondrial DNA replication, were found in severe, early-onset neurologic disorders, namely Alpers-Huttenlocher hepatopathic poliodystrophy, sensory-ataxia neuropathy with dysarthria and ophthalmoplegia, and spinocerebellar ataxia-epilepsy syndrome (Naviaux and Nguyen 2005).

Over the last decade, an increasing number of genes have been identified in association with mtDNA multiple deletions or depletion with variable phenotypes hallmarked by syndromic CPEO, encephalomyopathy, and cardiomyopathy. The vast majority of them involve proteins with a role in the mtDNA replisome (POLG and 2, TWNK, DNA2, MGME1) or dNTP supply for mtDNA synthesis (TP, TK2, DGUOK, RRM2B, SUCLA2, SUCLG1). A novel category of proteins involved in accumulation of mtDNA multiple deletions is represented by OPA1 and MFN2, which are part of the complex machinery regulating mitochondrial dynamics, specifically mitochondrial fusion; and paraplegin and AFG3L2, which play an important role in the protein quality control of mitochondria.

## Clinical features

Autosomal disorders classified as defects of mtDNA maintenance due to disturbed nuclear-mitochondrial intergenomic communication can be associated with the accumulation of mtDNA large-scale rearrangements (mtDNA multiple deletions) or by severe reduction of the mtDNA copy number (mtDNA depletion syndromes) (Spinazzola and Zeviani 2005). The most frequent clinical presentations (Table 1) are:

1. adult-onset encephalomyopathy, defined clinically by CPEO, genetically by autosomal dominant or recessive transmission, and molecularly by the presence of multiple deletions of mtDNA.
2. an autosomal recessive multisystem disorder known as mitochondrial neurogastrointestinal encephalomyopathy (MNGIE), characterized by combined accumulation of multiple deletions and partial depletion of mtDNA.
3. a spectrum of recessive neurologic syndromes ranging from typical infantile hepatopathic poliodystrophy (Alpers-Huttenlocher syndrome) to juvenile-onset sensory-ataxia neuropathy, dysarthria, and ophthalmoplegia (SANDO) to a combination of spinocerebellar ataxia and epilepsy (SCAE) with or without external ophthalmoplegia.
4. early-onset, organ-specific autosomal recessive syndromes associated with profound mtDNA depletion.

**Table 1:** Genes and phenotypes affecting mtDNA maintenance

Gene	mtDNA alteration	Inheritance	Main clinical phenotype	OMIM
*SLC25A4*	Multiple deletions	AD	ad/arCPEO	157640/258450
	Multiple deletions	AR	myopathy and cardiomyopathy	615418
	Depletion	AD	myopathy and cardiomyopathy	
*TWNK*	Multiple deletions	AD	adCPEO	609286
	Multiple deletions	AR	IOSCA	271245
	Depletion	AR	Alpers-like	
*POLG*	Multiple deletions	AD	adCPEO	157640
	Multiple deletions	AR	arCPEO	258450
	Depletion	AR	Alpers-Huntenlocher	203700
	Multiple deletions	AR	SANDO/SCAE	607459
*POLG2*	Multiple deletions	AD	adCPEO	610131
*TFAM*	Depletion	AR	Hepatocerebral syndrome	617156
*MGME1*	Multiple deletions	AR	arCPEO	615076
*DNA2*	Multiple deletions	AD	adCPEO	615156
*RNASEH1*	Multiple deletions	AR	arCPEO	616479
*RRM2B*	Multiple deletions	AD	adCPEO	613077
	Depletion	AR	myopathy and tubulopathy	612075
*TK2*	Depletion	AR	myopathy	609560
	Multiple deletions	AR	arCPEO	617069
*DGUOK*	Depletion	AR	Hepatocerebral syndrome	251880
	Multiple deletions	AR	Myopathy with or w/o CPEO	617070
	Multiple deletions	AR	lower motor neuron syndrome	
*MPV17*	Depletion	AR	Hepatocerebral syndrome	256810
	Multiple deletions	AR	arCPEO, leukoencephalopathy and parkinsonism	
*OPA1*	Multiple deletions	AD	DOA	165500
	Multiple deletions	AD	DOA plus	125250
*MFN2*	Multiple deletions	AD	DOA plus	608507
*SPG7*	Multiple deletions	AR	arCPEO and ataxia	602783
*AFG3L2*	Multiple deletions	AD	arCPEO and ataxia	604581
*TYMP*	Multiple deletions and depletion	AR	MNGIE	603041
*SUCLA2*	Depletion	AR	Hepatocerebral syndrome	612073
*SUCLG1*	Depletion	AR	Hepatocerebral syndrome	245400
*ABAT*	Multiple deletions	AR	Encephalomyopathy	613163
*FBXL4*	Depletion	AR	Encephalomyopathy	615471
*GFER*	Multiple deletions	AR	myopathy	613076

### Autosomal dominant chronic progressive external ophthalmoplegia (adCPEO)

CPEO is frequent in mitochondrial disorders. The clinical hallmark is extraocular muscle involvement; all patients have ptosis with limitation of eye movements. The first symptoms typically appear when patients are 20 to 40 years old. Generalized muscle weakness is frequently present. Additional features vary among families; they may include ataxia, sensorineural hearing loss, cataracts, hypogonadism, parkinsonism, and psychiatric abnormalities consisting of severe depression and avoidant personality. Dysphagia, dysphonia, weakness of facial muscles, and peripheral neuropathy may be prominent symptoms in some families. At rest, elevated levels of plasma lactate are detected only in severely affected patients. Symptoms seem to progress with age. Muscle biopsies show ragged-red fibers due to the subsarcolemmal accumulation of abnormal mitochondria. In addition, the histochemical reaction for cytochrome *c* oxidase (COX, complex IV) is decreased or absent in scattered fibers, and neurogenic changes may also be observed. Biochemically, the activities of mtDNA-related respiratory complexes (complex I, III, IV, and V) in muscle homogenate can range from normal to about 50% of the controls' mean (Servidei et al 1991). Presymptomatic patients appear normal but often have laboratory, electrophysiological, morphological, and biochemical features of a subclinical mitochondrial encephalomyopathy. Autosomal dominant optic atrophy has been reported in association with CPEO and multiple mtDNA deletions. Additional signs included deafness, ataxia, axonal sensory-motor polyneuropathy, and mitochondrial myopathy with cytochrome *c* oxidase negative and ragged red fibers (Yu-Wai-Man et al 2010).

### Autosomal recessive chronic progressive external ophthalmoplegia (arCPEO)

Multiple deletions of mitochondrial DNA have been reported in numerous sporadic CPEO cases or in families in which CPEO was clearly transmitted as a recessive trait (Lamantea et al 2002).

### Mitochondrial neurogastrointestinal encephalomyopathy (MNGIE)

This is an autosomal recessive disease characterized by the unusual combination of six features: (1) progressive external ophthalmoplegia, (2) severe gastrointestinal dysmotility, (3) cachexia, (4) peripheral neuropathy, (5) diffuse leukoencephalopathy on brain MRI, due to altered blood–brain barrier, and (6) evidence of mitochondrial dysfunction (histological, biochemical, or genetic abnormalities of the mitochondria) (Hirano et al 1994). The mean age at onset is 19 years, and the mean age at death is 37.6 years. Gastrointestinal manifestations comprise the most prominent and debilitating feature; painful gastrointestinal dysmotility may cause gastroparesis, frequent diarrhea, and intestinal pseudo-obstruction. Skeletal muscle biopsies reveal neurogenic changes and occasional ragged-red and cytochrome *c* oxidase-deficient fibers, reflecting the neuropathy and mitochondrial myopathy. The peripheral neuropathy is predominantly demyelinating, but electrophysiological data have also shown evidence of axonopathy in about one half of the patients. In the gastrointestinal system, histological studies have revealed abnormalities of both the intestinal smooth muscle and the enteric nervous system, thus accounting for the severe gastrointestinal problems. In particular, studies of postmortem tissues from MNGIE patients revealed a selective, profound mtDNA depletion, and marked atrophy of the external layer of the muscularis propria of the small intestine, consistent with a visceral myopathy leading to the prominent gastrointestinal involvement typical of this disorder (Giordano et al 2006, 2008). Later-onset and longer survival MNGIE patients have been reported (Marti et al 2005).

### Alpers-Huttenlocher hepatopathic poliodystrophy; sensory-ataxia neuropathy, dysarthria and ophthalmoplegia (SANDO); and spinocerebellar ataxia-epilepsy (SCAE)

A number of recessive syndromes have been associated with qualitative or quantitative mtDNA abnormalities. These syndromes are all characterized by an association with recessive mutations of *POLG*, the master gene of mtDNA replication. Alpers-Huttenlocher hepatopathic poliodystrophy is an early onset, fatal disease, characterized by hepatic failure and intractable seizures, evolving into epilepsia partialis continua and global neurologic deterioration. The liver dysfunction is usually progressive as well, evolving from microvesicular steatosis with bile duct proliferation into cirrhosis. Childhood-onset or juvenile-onset autosomal recessive, progressive sensory-ataxic syndromes, with or without epilepsy, have been reported, more frequently in families from Northern European countries. The association of sensory ataxic neuropathy with dysarthria and ophthalmoplegia (SANDO) defines some of these families, but in other families, cerebellar signs, myoclonus, and seizures are additional prominent findings. The use of valproate to control epilepsy is contraindicated in these patients because of their exquisite sensitivity to valproate hepatotoxicity, which may lead to fatal acute hepatic failure (Davidzon et al 2005; Gauthier-Villars et al 2001; Horvath et al 2006; Naviaux and Nguyen 2005; Naviaux et al 1999; Tzoulis et al 2006).

A tentative classification of these entities has been proposed (Copeland and Longley 2014), according to the following nomenclature: Alpers-Huttenlocher hepatopathic poliodystrophy (AHS), childhood myocerebrohepatopathy spectrum (MCHS), myoclonic epilepsy myopathy sensory ataxia (MEMSA), and the ataxia neuropathy spectrum (ANS). It is however important to stress that these entities constitute a continuous spectrum of syndromes with overlapping features (Fig. 2), with the exception perhaps of AHS which has rather specific clinical and neuroimaging hallmarks.

### Mitochondrial DNA depletion syndromes

In contrast to other types of mtDNA defects, mtDNA depletion syndrome is a quantitative abnormality: there is paucity of mtDNA , but the remaining mtDNA does not harbor any mutations or rearrangements. Mitochondrial DNA depletion syndrome is transmitted as an autosomal recessive trait and is clinically and genetically heterogeneous.

Some children present with myopathy, others with liver failure in infancy, and some with multisystem involvement. Consistent with the different phenotypes, mtDNA depletion may affect either a specific tissue (most commonly muscle or liver and brain) or multiple organs, including heart, brain, and kidney.

### Myopathic form

Typically, affected children are born after an uncomplicated pregnancy, although decreased fetal movements are noted in some cases. A few patients had arthrogryposis and clubfeet, but facial dysmorphic features are rare. The patient usually presents in the first year of life with feeding difficulty, failure to thrive, hypotonia, weakness, and occasionally progressive external ophthalmoplegia. Death is usually due to pulmonary insufficiency and infections, but some patients survive into their teens (Moraes et al 1991; Tritschler et al 1992). The clinical spectrum has now expanded to include spinal muscular atrophy type 3-like presentation, rigid spine syndrome, severe muscle weakness with marked dystrophic alterations, encephalopathy, and seizures (Galbiati et al 2006), and a milder myopathic phenotype without motor regression and with longer survival (Oskoui et al 2006). mtDNA depletion in muscle has been reported in patients with early-onset myopathy, lactic acidosis, and renal proximal tubulopathy with nephrocalcinosis (Bourdon et al 2007). In patients with earlier onset and rapid courses, all muscle fibers have little or no immunoreactivity to DNA antibodies in mitochondria and are cytochrome *c* oxidase-deficient, whereas in later-onset patients the pattern of involvement is mosaic; some fibers appear normal, whereas others lack both COX activity and mtDNA. Proliferation of mitochondria (ragged-red fibers) is not a consistent feature, but their number can increase with age. Biochemical defects of all complexes containing mtDNA-encoded subunits are always present in muscle mitochondria. Patients with mtDNA depletion in muscle tend to have elevated serum creatine kinase levels, ranging from 2 to 30 times the upper limit of normal. This is an important clue for the diagnosis because increased serum creatine kinase is relatively uncommon in patients with other mitochondrial myopathies.

### Encephalomyopathic forms

Two forms have been reported, both caused by a block of succinyl-CoA lyase activity in the Krebs cycle. The first is characterized by high lactate in blood, severe psychomotor retardation with muscle hypotonia, impaired hearing, and generalized seizures followed by knee and hip contractures, finger and limb dystonia, and mild ptosis. Brain MRI is suggestive of Leigh syndrome. Moderate mtDNA depletion (about 32%) has been documented in skeletal muscle (Carrozzo et al 2007; Elpeleg et al 2005; Ostergaard et al 2007a). Mutations in the gene encoding the ATP-dependent succinyl-CoA synthase (SCS) activity, SUCLA2, are responsible for this form. The second, extremely severe form is due to mutations in SUCLG1, the gene encoding the GTP-dependent isoform SUCLG1 (see below) and is associated with combined muscle and liver mtDNA depletion, dysmorphic features, connatal lactic acidosis, and death in the first days of life (Ostergaard et al 2007b). Both syndromes are hallmarked by methylmalonic aciduria, which accumulates because of impaired conversion into succinyl-CoA of propionyl-CoA, derived from the beta oxidation of odd-number fatty acids. A large cohort of patients with mutations in SUCLA2 and SUCLG1 has been recently described (Carrozzo et al 2016), showing significantly longer survival in SUCLA2 patients compared to SUCLG1 patients. Hypertrophic cardiomyopathy and liver involvement were exclusively found in patients with SUCLG1 mutations, whereas epilepsy was much more frequent in patients with SUCLA2 mutations compared to patients with SUCLG1 mutations.

Finally, patients with mutations in the *ABAT* gene, encoding 4-aminobutyrate aminotransferase have elevated levels of GABA along with severe psychomotor retardation, intractable seizures, hypotonia, and hyperreflexia, associated with profound mtDNA depletion (Besse et al 2015). ABAT, besides its role in GABA biosynthesis, encodes another component of the SCS.

The mechanism of mtDNA depletion in mutations of the SCS components is unclear but could be related to the physical interaction of SCS with nucleoside diphosphate kinase, NDPK, an enzyme involved in the salvage pathway of mitochondrial nucleotides.

### Hepatocerebral form

In addition to patients with Alpers-Huttenlocher syndrome, hepatic mtDNA depletion is seen in some infants with liver failure (Mazziotta et al 1992). Onset of symptoms is between birth and 6 months; death usually occurs within 1 year of age. The most common symptoms and signs include persistent vomiting, failure to thrive, hypotonia, and hypoglycemia associated with progressive neurologic symptoms. Histological changes on liver biopsy include fatty degeneration, bile duct proliferation, fibrosis, and collapse of lobular architecture. Generalized reduction in COX histochemistry and decreased mitochondrial respiratory chain enzyme activities were found in the liver of a few patients. A variant form of hepatocerebral mitochondrial DNA depletion syndrome affects the Navajo people with a prevalence of 1 in 1600 live births, hence the term Navajo neurohepatopathy (Vu et al 2001).

## Molecular genetics

**Fig. 1:**
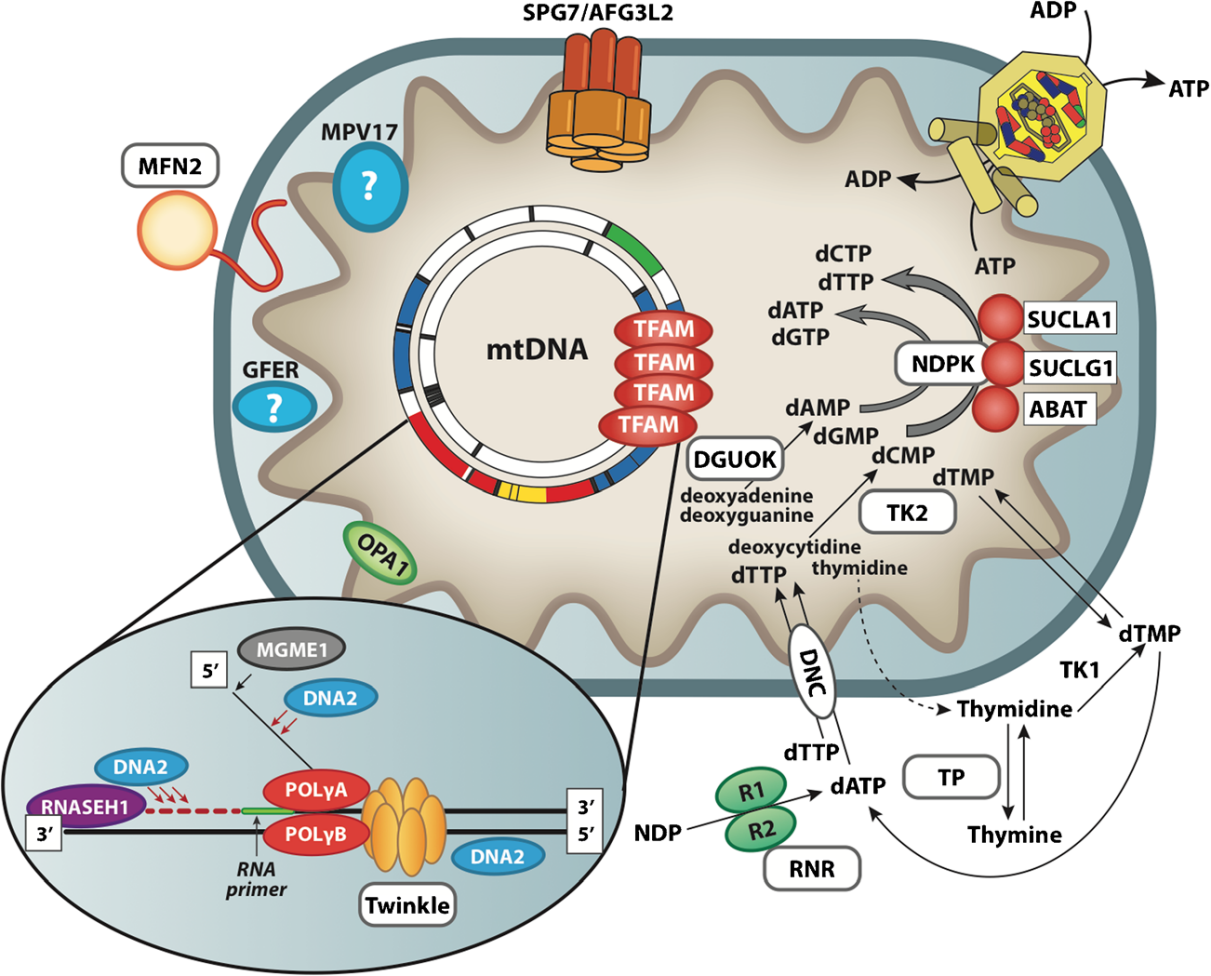
Pathways involved in mtDNA maintenance

Numerous genes have been associated with syndromes caused by mtDNA instability (Fig. 1) but a substantial fraction of these conditions remain genetically undefined.

### ANT1

Encoded by *SLC25A4*, ANT1 is the muscle-specific isoform of the mitochondrial adenine nucleotide translocator, and it is also expressed in heart and brain. Several mutations in *SLC25A4* have been linked to mitochondrial disorders and fall into two distinct clinical phenotypes. The first is caused by several single heterozygous mutations, and manifests as adCPEO (Deschauer et al 2005; Kaukonen et al 2000; Komaki et al 2002; Napoli et al 2001; Siciliano et al 2003). The second consists of a relatively benign mitochondrial myopathy and cardiomyopathy phenotype that presents in childhood or early adulthood and is characterized by fatigue and exercise intolerance, associated with recessive null mutations (Echaniz-Laguna et al 2012; Korver-Keularts et al 2015; Palmieri et al 2005; Strauss et al 2013). From the molecular point of view, both dominant and recessive mutations lead to the accumulation of multiple mtDNA deletions, and their clinical course is relatively benign. However, de novo dominant mutations in *SLC25A4* have recently been identified by whole exome sequencing in seven patients presenting with profound congenital hypotonia and muscle weakness, leading to death in the neonatal period. Profound mtDNA depletion and impairment of ATP transport capacity is the hallmark of this early onset form of ANT1-related disease (Thompson et al 2016). However, the reason why either recessive or dominant mutations in a monomeric protein (Bamber et al 2007) give rise to such a wide spectrum of clinical and molecular phenotypes is still unknown.

### TWNK

**Fig. 2:**
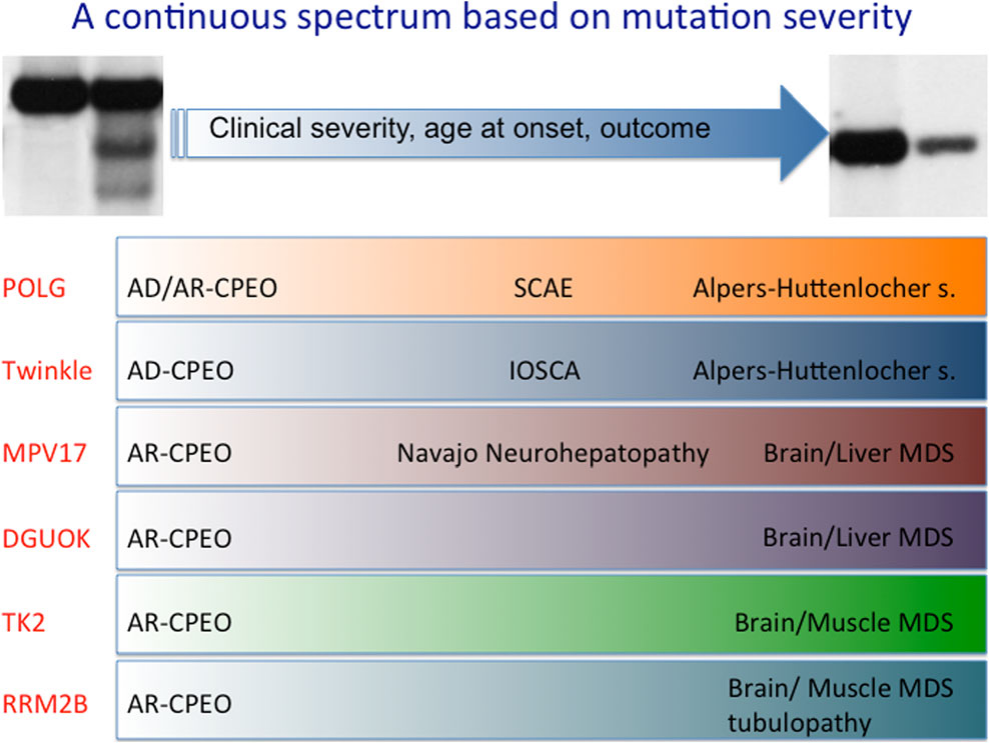
Scheme representing the continuous spectrum of clinical phenotypes associated with mutations in mtDNA-maintenance-related genes

This gene (previously designated as C10orf2) encodes Twinkle, the mitochondrial DNA and RNA helicase involved in replication of the mitochondrial genome. Heterozygous mutations in this gene are responsible for adCPEO (Spelbrink et al 2001) with accumulation of multiple mtDNA deletions. Clinical presentations include CPEO, often associated with proximal muscle and facial weakness, dysphagia and dysphonia, mild ataxia, and peripheral neuropathy. Symptoms are much more severe in a few homozygous mutant patients described in consanguineous families. A specific, recessive *TWNK* mutation is responsible for infantile onset spinocerebellar ataxia, IOSCA (Nikali et al 2005), which is part of the Finnish disease heritage. Onset usually is between 1 and 2 years of age. Patients suffer from a combination of ataxia, athetosis, areflexia, muscle hypotonia, and severe epilepsy. Other features such as ophthalmoplegia, hearing loss, and optic atrophy appear later in the disease course. Some patients show reduced mental capacity, and hypergonadotropic hypogonadism may occur in girls. Morphologic studies reveal sensory axonal neuropathy and progressive atrophy of the cerebellum, brainstem and spinal cord (Koskinen et al 1994). Besides infantile onset spinocerebellar ataxia, an Alpers-like clinical phenotype has also been associated with recessive mutations in *TWNK* (Fig. 2) (Hakonen et al 2007; Sarzi et al 2007).

### Pol-gamma mutations

The mitochondrial DNA polymerase (pol-gamma) is essential for mitochondrial DNA replication and proofreading-based repair. It is composed of a 140-kDa catalytic subunit (pol-[gamma]A) and a 55-kDa accessory subunit (pol-[gamma]B), which functions as a DNA binding factor, increasing the processivity of the polymerase holoenzyme. The holoenzyme works as an A_1_B_2_ heterotrimer. The catalytic subunit is encoded by *POLG* on chromosome 15q25, whereas the accessory subunits are coded by *POLG2* on chromosome 17q. Mutations of *POLG* are a major cause of human mitochondrial disease. So far more than 100 mutations in pol-gamma have been reported (http://tools.niehs.nih.gov/polg). This gene is the most frequent cause of adCPEO (50% of the cases in our series) (Lamantea et al 2002; Van Goethem et al 2001). In addition to CPEO, prominent features are severe dysphagia and dysphonia and, occasionally, a movement disorder including parkinsonism, cerebellar dysfunction, or chorea (Luoma et al 2004). Mental retardation, hypogonadism (including precocious menopause), and gastrointestinal dysmotility may be additional findings (Filosto et al 2003; Luoma et al 2004). The severity of the syndromes varies in relation to the type of mutation.

Recessive mutations of *POLG* are also responsible for a variety of other syndromes, including most of the autosomal recessive CPEO cases (Lamantea et al 2002) and the apparently sporadic CPEO cases associated with the accumulation of multiple mt DNA deletions (Agostino et al 2003). Recessive *POLG* mutations have been described in Alpers-Huttenlocher syndrome (Davidzon et al 2005; Ferrari et al 2005; Naviaux and Nguyen 2004) associated with mtDNA depletion. Mutations in *POLG* are also responsible for SANDO (Van Goethem et al 2003) and SCAE syndromes identified in Northern Europe (Van Goethem et al 2004; Winterthun et al 2005; Horvath et al 2006). In several instances, the same *POLG* mutations have been reported in these different syndromes, suggesting that they form a continuum of “*POLG*-related,” severe, recessive disorders (Fig. 2). Two mutant alleles determining amino acid changes in the spacer region of the pol-gammaA protein (p.Ala467Thr and p.Trp748Ser) are often recurrent in these conditions, which may help the diagnostic workout in suspected cases.

A few heterozygous dominant mutations have been identified in *POLG2*, in patients with adult-onset CPEO, cardiac conduction defect, and increased creatine kinase (Longley et al 2006; Walter et al 2010; Young et al 2011).

### TFAM

A homozygous mutation in TFAM has been found in two siblings with a severe neonatal hepatic syndrome (Stiles et al 2016) and profound mtDNA depletion in liver and skeletal muscle. The mutation (c.533C > T) affects proline 178 (p.Pro178Leu), which is important for the interaction with the mtDNA minor groove. As a consequence of the mutation, the interaction of TFAM with mtDNA is impaired, TFAM undergoes degradation and nucleoids are reduced in number and aggregate to form perinuclear clusters.

### MGME1, DNA2, RNASEH1

In addition to the main constituents of the mitochondrial replisome, i.e., Pol-gamma A and B and the helicase Twinkle, several genes have been identified which encode proteins involved in maturation of the newly synthesized mtDNA strands and may also take part in some mechanisms of mtDNA repair, notably long-patch base-excision repair (LP-BER). Some of these gene products, such as the DNA flappase MGME1, are exclusively localised within mitochondria. Others have a double localization, in both the nucleus and mitochondria. For instance DNA2 and FEN1, which are also involved in the processing of 5′ flap structures occurring in DNA replication and LP-BER, and RNAseH1, which specifically digests the RNA component of RNA/DNA hybrids, formed during RNA priming of DNA templates. Mutations in *MGME1* (Kornblum et al 2013), *DNA2* (Ronchi et al 2012) and *RNASEH1* (Reyes et al 2015) have recently been identified in patients with PEO and accumulation of multiple mtDNA deletions. Whilst *MGME1* and *RNASEH1* mutations are inherited as recessive traits, *DNA2* mutations seem to be dominant. The onset is usually in adulthood, more rarely in childhood, and PEO is frequently complicated by prominent weakness of the respiratory muscles, leading to ventilatory insufficiency, ataxia with cerebellar atrophy and additional signs indicating the involvement of the central and peripheral nervous systems.

### *RRM2B*, *TK2, DGUOK*, and *MPV17*

These genes were initially associated with mtDNA depletion syndromes but allelic variants were later linked to adult-onset disorders associated with mtDNA multiple deletions, in most cases CPEO syndromes (Fig. 2).

*RRM2B* encodes the small (regulatory) subunit of p53-inducible ribonucleotide reductase, involved in the de novo conversion of ribonucleoside diphosphates into the corresponding deoxyribonucleoside diphosphates. Notably, this enzyme is localized in the cytosol, not in mitochondria, but in non-proliferating tissues it plays a major role in supplying deoxynucleotides for mtDNA replication. Heterozygous mutations in *RRM2B* are a prominent cause of adCPEO (Fratter et al 2011; Tyynismaa et al 2012). Recessive mutations in *RRM2B* are typically found in infantile cases of myopathic mtDNA depletion syndrome with renal proximal tubulopathy (Bourdon et al 2007), but a single family was associated with a recessive form of CPEO (Takata et al 2011). A clear correlation between the clinical phenotype and the underlying genetic defect was found in a relatively large cohort of patients (Pitceathly et al 2012). Myopathy, bulbar dysfunction, and fatigue were prominent symptoms, often associated with sensorineural hearing loss and gastrointestinal disturbance. Severe multisystem neurological disease was associated with recessively inherited compound heterozygous mutations with a mean age of disease onset at 7 years. Dominantly inherited heterozygous mutations were associated with a milder predominantly myopathic phenotype.

TK2 is the mitochondrial deoxyribonucleoside kinase that phosphorylates thymidine, deoxycytidine, and deoxyuridine. Recessive mutations in *TK2* have been associated with both the myopathic form of mtDNA depletion syndrome and CPEO with mtDNA multiple deletions (Tyynismaa et al 2012). Multiple deletions have also been reported in adult myopathic patients with slowly progressive weakness and respiratory failure (Alston et al 2013; Behin et al 2012; Paradas et al 2013).

Similarly, recessive mutations in *DGUOK*, encoding the mitochondrial deoxyguanosine kinase, were initially found in association with the severe infantile hepatocerebral form of mtDNA depletion. Later, exome sequencing in adult patients revealed the presence of *DGUOK* mutations associated with a spectrum of clinical phenotypes ranging from mitochondrial myopathy with or without CPEO, recurrent rhabdomyolysis, and adult-onset lower motor neuron syndrome with mild cognitive impairment (Ronchi et al 2012). These patients had accumulation of mtDNA multiple deletions in skeletal muscle.

Finally, mutations in *MPV17*, encoding a small protein of the inner mitochondrial membrane of unknown function, were originally associated with hepatocerebral mtDNA depletion (Spinazzola et al 2006), and later found in patients with mtDNA multiple deletions and recessive adult-onset mitochondrial myopathy with CPEO, parkinsonism, and leukoencephalopathy (Blakely et al 2012; Garone et al 2012a, b).

### *OPA1* and *MFN2*

*OPA1* is a dynamin-like GTPase located in the inner mitochondrial membrane involved in mitochondrial fusion, cristae organization, and control of apoptosis. *OPA1* is linked to non-syndromic autosomal dominant optic atrophy (DOA), a condition characterized by slowly progressive visual loss starting in childhood, first described by the Danish ophthalmologist Paul Kjer in 1959. However, a few missense mutations, clustered in the GTPase domain, are responsible for a non-syndromic autosomal dominant optic atrophy “plus” syndrome, consisting of a combination of DOA with CPEO, peripheral neuropathy, ataxia, deafness (Amati-Bonneau et al 2008; Hudson et al 2008), and multiple sclerosis-like illness or spastic paraplegia (Yu-Wai-Man et al 2010). These patients have ragged-red and COX negative muscle fibers, with paracrystalline inclusions filling abnormally shaped mitochondria. In two large Italian families with autosomal dominant OPA1 mutations, adCPEO was associated with parkinsonism in several patients, whereas symptomatic optic atrophy was absent in most of the affected individuals (Carelli et al 2015). Remarkably, the “OPA1-plus” patients harbor multiple mtDNA deletions and OXPHOS deficiency in their skeletal muscle (Lodi et al 2011). The mechanisms leading to the accumulation of multiple mtDNA deletions in this condition are still unknown but indicate that mitochondrial shape and mtDNA integrity are linked, possibly through a mechanism controlling the structure and function of nucleoids (Zeviani 2008).

Interestingly, a single family with DOA plus phenotype without CPEO has been reported in association with a *MFN2* heterozygous missense mutation (c.629A > T, p.D210V); these patients showed accumulation of mtDNA multiple deletions in skeletal muscle (Rouzier et al 2012).

Thus, impaired fusion due to mutations in both *OPA1* and *MFN2* proteins may lead to mtDNA instability and depletion through novel mechanisms, which are still under investigation.

### SPG7

*SPG7* encodes for paraplegin, a component of the m-AAA protease, an ATP-dependent proteolytic complex of the mitochondrial inner membrane that degrades misfolded proteins and regulates ribosome assembly (Nolden et al 2005). Originally associated with autosomal recessive spastic paraplegia (Casari et al 1998), *SPG7* recessive mutations have later been found in patients with CPEO and mtDNA multiple deletions (Pfeffer et al 2014; Wedding et al 2014). Typically, the clinical phenotype develops in mid-adult life with either CPEO/ptosis and spastic ataxia, or a progressive ataxic disorder. The mechanism leading to multiple deletions remains unclear.

### AFG3L2

Two patients with indolent ataxia and CPEO with two different heterozygous mutations in *AFG3L2* were recently identified (Gorman et al 2015). *AFG3L2* encodes another subunit of m-AAA protease which interacts with paraplegin, and was originally associated with spinocerebellar ataxia. Also in this case, the mechanism leading to mtDNA instability is unknown.

### Thymidine phosphorylase mutations in MNGIE

Mapping of the MNGIE trait on chromosome 22q13.32-qter (Hirano et al 1998) has led to the identification of mutations in the gene encoding thymidine phosphorylase (*TYMP*) as the cause of the disease (Nishino et al 1999). Thymidine phosphorylase is involved in the catabolism of pyrimidines by promoting the phosphorolysis of thymidine into thymine and deoxyribose-phosphate. Defects of thymidine phosphorylase result in systemic accumulation of its substrates—thymidine and deoxyuridine (Marti et al 2004; Spinazzola et al 2002). In vitro studies have demonstrated that excess of thymidine and deoxyuridine leads to deoxynucleotide pool imbalance (Ferraro et al 2005), which in turn can cause mtDNA instability (Pontarin et al 2006). This is reflected by the molecular phenotype of mitochondrial neurogastrointestinal encephalomyopathy, which is characterized by both multiple deletions and partial depletion of muscle mtDNA.

## Pathogenetic considerations

The CPEO phenotype is commonly associated with single mtDNA deletions in sporadic patients (Spinazzola and Zeviani 2005). A pathogenetic role of multiple mtDNA deletions in autosomal dominant or recessive CPEO is supported by the evidence of tight segregation of the molecular lesions with the onset and severity of the disease. For instance, Lamantea and colleagues have reported that, in a series of *POLG*-positive CPEO families, homozygous individuals appeared more severely affected and showed the presence of much higher amounts of multiple mtDNA deletions in muscle than their heterozygous relatives (Lamantea et al 2002). Moslemi and colleagues have demonstrated close correlation between the accumulation of deletions and the segmental ragged-red cytochrome *c* oxidase-negative transformation of muscle fibers (Moslemi et al 1996). These authors showed that within a single cytochrome *c* oxidase-deficient muscle fiber segment, only one single deletion could be detected. However, different deletions were identified in different segments. These results indicate clonal expansion of a single deleted mtDNA in each cytochrome *c* oxidase-deficient muscle fiber segment. A two-hit mechanism can, therefore, be hypothesized, consisting of the combination of a nuclear factor that somehow predisposes to mtDNA deletions, followed by clonal expansion of each deleted mtDNA molecule in muscle and other stable tissues (Moslemi et al 1996). Deletions are absent in cultured fibroblasts, peripheral blood cells, and cultured myoblasts but can be detected in stable tissues, including (besides skeletal muscle) brain, heart, and in lesser amount, kidney, and liver. Disturbances of the nucleotide pool available for mtDNA replication, as well as abnormalities in either the mitochondrial helicase or mtDNA polymerase, are likely to affect the rate or process of DNA replication, which could ultimately lead to the exaggerated production of rearranged mtDNA molecules (Graziewicz et al 2004). In addition to large-scale rearrangements, increased frequency of mtDNA point mutations have been reported in MNGIE (Nishigaki et al 2003). Several somatic mutations, mostly T > C transitions preceded by 5′-An sequences, were scattered throughout the mtDNA molecule of tissues and cultured cells from MNGIE patients. Some mutations were clearly pathogenic, as they predict loss or abnormal function of mtDNA-encoded proteins or tRNA. The accumulation of these mutations is likely to be due to next-nucleotide effects and dislocation mutagenesis, as a consequence of increased levels of mitochondrial deoxy-thymidine and deoxy-uridine pools (Nishigaki et al 2003).

Finally, the mtDNA damage caused by *POLG* mutations in Alpers-Huttenlocher syndrome, SANDO, and SCAE syndrome is unclear. Depletion of mtDNA in critical organs (liver and brain) has been demonstrated in a few cases (Ferrari et al 2005; Tzoulis et al 2014), which may explain the early onset and severity of these conditions.

Balance and control of the mitochondrial deoxynucleotide pools are essential for the maintenance of mtDNA integrity and copy number. Perturbation of this homeostatic control, as determined by defects of dGK and TK2, and possibly of thymidine phosphorylase, RRM2B, and ANT1 as well, can lead to mtDNA depletion or multiple deletions. In particular, dGK and TK2 are involved in the salvage pathways of mitochondrial deoxynucleotides, which constitute the major source of mtDNA precursors in stable tissues such as liver, brain, and muscle. Although a role for MPV17 has been proposed in the cellular response to metabolic stress and maintenance of nucleotide pools (Dalla Rosa et al 2016; Spinazzola et al 2006), its function remains largely unknown.

The identification of *OPA1* mutations as a cause of mtDNA multiple deletions in skeletal muscle, which is now extended to the cognate mitochondrial fusion protein MFN2 (Rouzier et al 2012), points to the role played by mitochondrial network dynamics in mtDNA maintenance (Yu-Wai-Man and Chinnery 2012; Zeviani 2008). Impaired mitochondrial fusion in cells and in recombinant mouse models is indeed associated with mtDNA instability and worsening of the phenotype effects of mtDNA mutations (Chen et al 2010). Likewise, multiple deletions associated with mutations in *SPG7* (paraplegin) and *AFG3L2* indicate a causative association between mtDNA instability and impaired protein quality control in mitochondria.

## Additional genes

The mechanisms leading to mtDNA instability are rather controversial for several of the genes associated with the disease.

For instance, *SUCLA1* and *SUCLG1* encode for components of the Krebs cycle and their role in the last step of the mitochondria dNTPs salvage pathway, because their association with NDPK, has been postulated but not conclusively proven. Similarly, *ABAT* is involved in GABA biosynthesis but also interacts with SCS and has been proposed to have a role in dNTP salvage pathway.

Another gene with an unclear role in mtDNA maintenance is *FBXL4*. This gene has been recently associated with early onset encephalopathy with mtDNA depletion (Bonnen et al 2013; Gai et al 2013). *FBXL4* encodes for F-box and leucine-rich repeat 4 protein targeted to the intermembrane space of mitochondria. Although pathogenic mutations are associated with substantially decreased mtDNA content and mitochondrial respiratory chain deficiency in muscle and fibroblasts, its role in mtDNA maintenance is still unclear.

Finally, a homozygous mutation in the human *GFER* gene, coding for a sulfhydryl oxidase (DRS) of the mitochondrial intermembrane space, has been reported in an inbred Moroccan family (Di Fonzo et al 2009). Three siblings were affected by congenital cataract, progressive muscular hypotonia, sensorineural hearing loss, and developmental delay. Muscle biopsy showed scattered COX deficiency and mtDNA multiple deletions. DRS is a protein involved in one of the mitochondrial protein import pathways. The pathogenic link between DRS mutation and mtDNA multiple deletions is presently unknown.

## Concluding remarks

The development of next-generation sequencing (NGS) technologies (whole exome sequencing and whole genome sequencing) and their use in the diagnostic workout for mitochondrial disease has had a tremendous impact on the pace of gene discovery in recent years, and a huge set of new genes have indeed been associated with either mtDNA multiple deletions or mtDNA depletion. Nevertheless, a substantial fraction of the cases associated with mtDNA instability remains unsolved. This may be due to a number of reasons. First, we now know that genes encoding proteins whose function is not directly related to mtDNA replication or repair, such as OPA1, MFN2, FBXL4, can affect mtDNA metabolism. This consideration requires a substantial expansion of the territory of gene hunting, well beyond that of the “usual suspects”. Second, mtDNA defects can be due to sporadic, heterozygous mutations, affecting singleton cases, as in the case of the recent identification of lethal de novo mutations in ANT1 (Thompson et al 2016). These cases can escape detection, particularly if the screening does not include parents and direct relatives of the proband. Third, the mutation can affect deep intronic regions, and may escape detection if ad hoc gene panels, or whole exome sequencing, WES, are carried out or are considered a benign variant. Fourth, in case of sporadic patients, oligo- or multi-genic inheritance cannot be excluded, which complicates the diagnostic work-up. Fifth, the expression of a pathological phenotype may depend on dual or multiple mechanisms, for instance the mtDNA haplogroup, the gender, or behavioral habits. Finally, in several cases the function of the culprit gene is unknown, and the attribution of a pathogenic role to these “unknown factors” may not be obvious. The accumulation of more WES and whole genome sequencing, WGS, data in the human population and the systematic use of trio sequencing will possibly reduce the gap between the biochemical and genetic diagnosis, not only in the syndromes associated with mtDNA instability but in mitochondrial medicine as a whole.

A tissue biopsy, typically muscle, remains the gold standard for the diagnosis of mtDNA instability syndromes. However, in an increasing number of cases the syndromic characterization is specific enough to consent the screening of suspected genes, by ad hoc gene panels or WES, before or alternative to the investigation of muscle biopsy. Muscle and, in specific cases, liver biopsies should be performed in the routine analysis for mitochondrial disease when the diagnosis cannot be confirmed by direct targeted DNA testing.

Functional in vitro assays in tissue (typically muscle) have been the mainstay of diagnosis of mitochondrial disorders, especially prior to the recent advances in genomics. Functional assays remain important measures of mitochondrial function. All of the mitochondrial disease guidelines and diagnostic criteria developed prior to the recent advances in genetic techniques and understanding include results of such biochemical studies to help establish a mitochondrial disease diagnostic workup (Nishigaki et al 2003; Nishino et al 1999; Nolden et al 2005; Oskoui et al 2006; Ostergaard et al 2007a).

These tests evaluate the various functions of the mitochondrial ETC or respiratory chain. Functional assays include enzyme activities of the individual components of the ETC, measurements of the activity of respiratory chain complexes and ATP synthase, blue-native gel electrophoresis, measurement of the presence of various protein components within complexes and supercomplexes (achieved via western blots and gel electrophoresis), and consumption of oxygen using various substrates and inhibitors. In any case we recommend that these assays should be carried out in specialized centers, as they require ad hoc expertise and standardized, verified and complex technologies and diagnostic protocols.

As for the majority of mitochondrial disorders, no treatment is currently available for disease associated with mtDNA instability. However, it has been recently demonstrated in mouse models knockout for Tymp and knockin for the Tk2 H126N mutation that the supplementation of deoxyribonucleotides can effectively correct mtDNA depletion (Camara et al 2014; Garone et al 2014). Albeit preliminary, these new experimental approaches open the possibility for ad hoc therapies for diseases due to mtDNA instability.
